# Butterfly Female Proportion Increases in the Understorey During the Rainy Season in a Tropical Dry Forest

**DOI:** 10.3390/insects17101053

**Published:** 2026-10-13

**Authors:** Camila Montesdeoca, Sebastián Mena, Jacqueline Rodriguez, Camila Rodríguez, Erika Páez, María F. Checa, Keith Willmott

**Affiliations:** 1 Museo de Zoología QCAZ Invertebrados, Escuela de Ciencias Biológicas, Pontificia Universidad Católica del Ecuador, Quito 170525, Ecuador; dsmenag@gmail.com (S.M.); camirodrigueze90@gmail.com (C.R.); erika_paezv@hotmail.com (E.P.); 2 Fundación Charles Darwin, Puerto Ayora 200350, Ecuador; jacqueline.rodriguez@fcdarwin.org.ec; 3 McGuire Center for Lepidoptera and Biodiversity, Florida Museum of Natural History, University of Florida, Gainesville, FL 32611, USA; kwillmott@flmnh.ufl.edu

**Keywords:** sex ratio, vertical stratification, tropical dry forest, seasonality, fruit-feeding butterflies, Nymphalidae

## Abstract

Vertical stratification is common among tropical forest butterflies. While the phenomenon has been broadly studied with respect to taxonomy and morphology, less is known about how seasons affect vertical distribution. Males and females often differ in morphology, reflecting divergent priorities such as mate location versus oviposition. No study has evaluated how seasons affect the vertical stratification of butterfly sexes, which is key to community dynamics. Here, we describe how the sex ratio of fruit-feeding butterflies varies between strata and seasons using bait trap data from a one-year study in a tropical dry forest in coastal Ecuador. We report a consistent seasonal shift in sex ratio across vertical strata for the ten most abundant species (89% of records). While the sex ratio was similar in the canopy and understory in the dry season, females became relatively more abundant in the understory and males relatively more abundant in the canopy during the rainy season. Firstly, new leaf flushes in young plants may attract females to the understory to search for oviposition sites. Secondly, males may spend more time in mate-location activities, in particular patrolling the forest canopy. These results highlight the complexity of drivers influencing vertical distribution in tropical dry forests.

## Simple Summary

Tropical forests are divided into vertical layers, such as the tree canopy and the shady understory, which offer different living conditions. While we know that different butterfly species prefer different forest layers, it is less clear how males and females divide their time between these microhabitats, especially as seasons change. We studied butterflies for a year in a tropical dry forest in Ecuador to understand how the sexes distribute themselves between the canopy and understory during the dry and rainy seasons. We found a striking seasonal shift. During the dry season, males and females were evenly mixed in the understory, likely seeking refuge from the hot, dry canopy. However, during the rainy season, the sexes separated: females became relatively more common in the lower understory, while males moved up into the canopy. We suggest that fresh plant growth in the understorey in the rainy season may attract females to lay eggs on new leaves, while males move upward to patrol for mates. These findings highlight how changing weather and breeding behaviors dictate where insects live within the forest and improve our understanding of the mechanisms driving variation in biological communities across small spatial scales.

## 1. Introduction

Neotropical seasonally dry forests are ecosystems defined by marked seasonal variation in rainfall and water availability. Such climates generate pronounced changes in vegetation structure and resource availability throughout the year [1]. Despite having high biodiversity and ecological importance, these forests are among the most threatened tropical ecosystems [2]. For example, the seasonally dry forests of western Ecuador have lost 27.04% of their original extent, through habitat loss and fragmentation due to factors such as agricultural expansion, unregulated cattle ranching, and human-induced fires, with the province of Manabí experiencing some of the highest deforestation rates in the region [3]. Our study site, Bosque Seco Lalo Loor (BSLL), located on the Pacific coast of Manabí, lies in a transition zone between wetter forests to the north and drier forests to the south, resulting in a mixture of species from these different environments [3].

Fruit-feeding butterflies are an important component of many Neotropical butterfly communities. In Neotropical forests, including dry forests, fruit-feeding butterfly assemblages are composed exclusively of nymphalids [4]. This guild is composed mainly of several Nymphalidae subfamilies, including Satyrinae, Biblidinae, Charaxinae, and some Nymphalinae [5]. Because these butterflies are attracted to fermented fruit, they are commonly sampled using baited traps, which have been widely used to study tropical butterfly communities and their vertical stratification [6].

Tropical forests show marked vertical stratification, where the canopy and understory differ not only in abiotic conditions such as light availability, temperature, and humidity, but also in resource distribution and biotic interactions. In tropical butterfly communities, biotic pressures such as predation and the mutualistic benefits of Müllerian mimicry are key drivers of habitat use, and they significantly shape vertical stratification within forests [6,7,8,9]. These environmental factors influence how butterflies use forest habitats and may result in males and females of the same species occupying different strata. For example, Ref. [10] found that while male butterflies outnumbered females in both forest strata in a Tanzanian forest, the male bias was markedly stronger in the canopy than in the understory, suggesting that the two sexes differ in their relative use of vertical strata. Seasonal changes in climate may also modify vertical distribution patterns, since temperature and humidity differ between dry and rainy seasons and may influence adult emergence and activity [11]. Understanding how the sex ratio of butterfly communities varies between forest strata and across seasons is therefore likely to provide insights into the factors driving the spatial and temporal dynamics of these communities as a whole.

Vertical stratification in fruit-feeding butterflies shows some phylogenetic conservatism, with patterns often differing among subfamilies. Most species of Charaxinae, Nymphalinae and Biblidinae are more abundant in the canopy, whereas those of Satyrinae and Morphinae are more abundant in the understory [12], with these patterns documented across multiple Neotropical sites [8,9,10]. Notably, vertical partitioning can also occur at the intraspecific level; for example, within the genus *Morpho*, males of canopy-dwelling species patrol at treetop heights using gliding flight, whereas females of the same species frequently utilize flapping flight in the understory where they search for oviposition sites [13].

Flight morphology further differs between strata; species with stout bodies and low wing-to-thorax ratios are more commonly associated with the canopy, whereas species with slender bodies and high wing-to-thorax ratios are more frequently found in the understory [14,15,16]. These morphological differences are thought to reflect adaptations in flight physiology and aerodynamic efficiency, such as the enhanced gliding performance documented in canopy-dwelling *Morpho* species [17], as well as chemical defense, predator avoidance, and potentially differing mate-locating strategies.

Sexual dimorphism is also widespread in Lepidoptera, with males and females frequently differing notably in body size, wing shape, and behavior [18]. Such dimorphism suggests different selective pressures on the two sexes and the possibility that they may also show different preferences for vertical position in the forest. In particular, males are likely mainly engaged in mate location and acquiring critical nutrients, and females in seeking oviposition sites. Females searching for host plants may be constrained to fly at the heights where their hostplants grow [19]. In contrast, males of many butterfly species use elevated terrain as mate-locating sites, congregating on hills and ridges where they are more likely to encounter receptive females, a behavior known as hilltopping [20]. Similarly, in some species, males occupy elevated positions in the forest canopy, from which they intercept females in flight and engage in territorial interactions with other males [10,21]. Microclimatic conditions may reinforce these behavioral differences and drive morphological divergence, as the canopy is generally warmer and more variable, whereas the understory is cooler and more stable [14].

Adult sex ratios in Lepidoptera are known to vary with environmental conditions. Males are typically more abundant than females, a pattern that has been attributed to higher mortality of recently emerged females [22]. In Brazilian Atlantic Forest fruit-feeding butterflies, five out of six abundant species showed male-biased sex ratios [23]. Seasonal climate changes are also linked with sex-specific responses. In the Satyrinae butterfly *Pararge aegeria*, individuals from different seasonal cohorts differ in body size and wing coloration between sexes [24]. In addition, larger butterfly species tend to have shorter flight periods than smaller species [11].

In summary, behavioral, morphological and physiological differences between adult male and female tropical forest butterflies all suggest the likelihood of differences in their vertical distribution, but these patterns have been little explored to date, and the impact of seasonality on vertical distribution across sexes remains unknown. In this study we therefore evaluated the vertical distribution of male and female fruit-feeding butterflies across forest strata and seasons in a Neotropical dry forest. We addressed two main questions: firstly, do sexes differ in their vertical distribution (is sex ratio different in the canopy and understory), and, secondly, how do sex ratios in each stratum change across dry and rainy seasons? Because sex ratio in each stratum is shaped by the simultaneous movement of both sexes, and because seasonal variation in microclimate, host plant phenology, and male mate-locating behavior may interact in complex and opposing ways, we present our findings as exploratory and discuss potential associated factors in the discussion.

## 2. Material and Methods

### 2.1. Study Area

Our study was conducted at Reserva Bosque Seco Lalo Loor (BSLL; 0°04′27″ S, 80°09′03″ W), a 180 ha private reserve located on the central coast of Ecuador within the Tumbesian biogeographic region, one of the world’s biodiversity hotspots [25]. According to the Köppen–Geiger climate classification, the area is characterized as a tropical savanna climate (Aw) [26], which corresponds locally to the Ecuadorian ecosystem classification of “Bosque semideciduo de tierras bajas de la Costa” [27]. This seasonally dry tropical forest is characterized by semi-deciduous vegetation and canopy heights up to 25 m. Dominant tree species include *Ceiba trichistandra*, *Bursera graveolens*, *Tabebuia chrysantha*, and *Geoffroea spinosa* [28]. The region experiences marked seasonality, with a rainy season from January to March and a prolonged dry season beginning in June [29].

### 2.2. Census Techniques

We performed standardised butterfly monitoring surveys in BSLL between the years 2009–2010, collecting specimens using Van Someren–Rydon bait traps in the canopy and the understory. Butterflies were sampled along two transects, each having eight sampling points where two traps were deployed at each point, one in the understory (~2 m above the ground) and one in the canopy (20–25 m above the ground). Thus, a total of 32 traps were established across 16 sampling points, with 16 traps baited with banana and 16 with shrimp. The sampling points were spaced at least 70 m apart [29].

Sampling was carried out over 13 months in 6-day sampling periods each month. Two types of baits were used: 2-day-old fermented mashed banana (fruit) and rotting shrimp that had been decomposing for 15–18 days (carrion). Previous studies in the Ecuadorian Amazon have shown that carrion bait can attract a greater number of butterfly species and individuals than fruit bait [30,31]. More recently, found that carrion baits, including shrimp, attracted substantially higher butterfly abundance than banana, while differences among the types of carrion bait were comparatively small. Shrimp was therefore selected as a consistent and effective carrion bait for our monitoring protocol [32].

Baits were alternated between adjacent sampling points, so that neighboring points had different baits, while the canopy and understory traps within the same point were baited with the same type. Traps were checked daily for butterflies. Because carrion baits are known to attract predominantly males, we restricted all analyses in this study to butterflies captured in fruit-baited traps to ensure a balanced representation of both sexes. Trapped butterflies were identified and collected, and specimens were deposited at the Museo de Invertebrados, Pontificia Universidad Católica del Ecuador (QCAZ-I). Butterfly collection was conducted under research permits issued by the Ministerio del Ambiente del Ecuador.

### 2.3. Seasonal Classification

Months were classified as dry or rainy based on monthly precipitation derived from the CHIRPS dataset (Climate Hazards Group InfraRed Precipitation with Station data; spatial resolution ~0.05°, ~5.5 km) [33]. To establish an objective threshold, we followed the Köppen climate classification for tropical dry forests, where a dry month is defined as having cumulative rainfall < 60 mm [27]. Based on this criterion, the dry season comprised June–December 2009 and June 2010 (8 months), whereas the rainy season spanned January–May 2010 (5 months); these two seasons were used as the main grouping variable in all subsequent analyses. To place the sampling period in a broader climatic context, we also compiled annual precipitation for 2005–2015 (Tables S1 and S2).

### 2.4. Statistical Analyses

All analyses were conducted in R 4.2.2 [34], and figures were generated with the package ‘ggplot2’ [35]. To ensure meaningful comparisons and reduce the influence of rarely observed taxa, we restricted the analyses to the ten most abundant species.

To describe temporal variation in abundance for each sex we calculated the proportion of females by month in each stratum. To classify sampling periods into seasons, monthly precipitation was derived from CHIRPS version 1.1 [33] by summing daily records per month. Differences in monthly precipitation between the dry and rainy seasons were evaluated using a Wilcoxon rank-sum test because sample sizes were small (n = 8 dry and n = 5 rainy months) and precipitation values were strongly skewed, violating the assumptions of parametric tests. These data were incorporated into temporal plots to visually compare changes in female proportions with seasonal precipitation patterns.

To study variation in the sex ratio of butterflies across forest strata (canopy and understory) and seasons, we fitted a binomial generalized linear mixed model (GLMM) [36] with a logit link, using the counts of females and males as the response variable. The logit link was chosen because it is the canonical link function for the binomial family, ensuring that predicted values remain bounded between 0 and 1, as expected for proportional data. Fixed effects included forest stratum (canopy and understory), season (dry and rainy), and their interaction (stratum × season). To account for interspecific variation in sex ratio, the variable ‘species’ was included as a random intercept. As a complementary analysis, we fitted binomial generalized linear models (GLMs) for each species using the function glm in the package stats of R [35]. As a complementary analysis, we fitted a binomial generalized linear model (GLM) including stratum, season, their interaction, and species as fixed effects, to explore species-specific patterns. This was done using the function glm in the package stats of R [34]. Predicted proportions and 95% confidence intervals (95% CIs) were obtained from the fitted models using the ‘ggeffects’ package [37]. Mixed models were fitted using ‘lme4’ [38]. Effect sizes were reported as odds ratios (ORs) with their corresponding 95% CIs.

## 3. Results

We captured a total of 2428 individuals from 54 species across seven subfamilies of Nymphalidae, with the community dominated by Charaxinae, Biblidinae, Nymphalinae, Satyrinae and Apaturinae. To evaluate temporal and vertical variation in sex ratio, we focused on the 10 most abundant species, which together represented 88.7% of total community abundance. These species were: *Archaeoprepona demophon* (Charaxinae), *Colobura dirce* (Nymphalinae), *Fountainea ryphea* (Charaxinae), *Hamadryas amphichloe* (Biblidinae), *Hamadryas amphinome* (Biblidinae), *Historis acheronta* (Nymphalinae), *Historis odius* (Nymphalinae), *Myscelia cyaniris* (Biblidinae), *Smyrna blomfildia* (Nymphalinae), and *Taygetis nympha* (Satyrinae) (Table 1).

**Figure 1:**
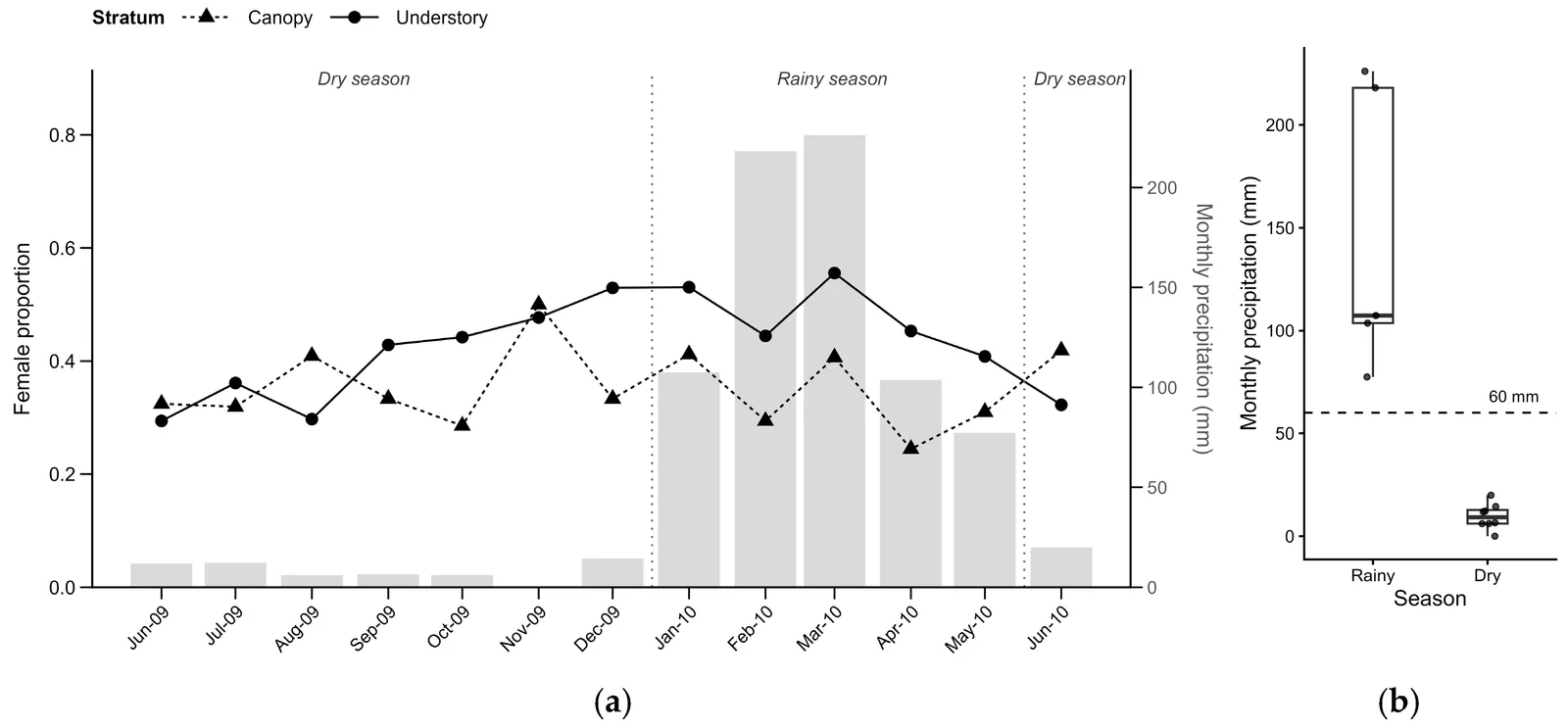
Seasonal variation in female proportion and precipitation. (**a**) Female proportion (females/total individuals) of fruit-feeding butterflies captured in banana-bait traps in the canopy and understory from June 2009 to June 2010. The gray bars represent monthly precipitation (mm), and the vertical dashed line indicates the transition between the dry and rainy seasons. (**b**) Distribution of monthly precipitation during the dry (n = 8 months) and rainy (n = 5 months) seasons. Boxplots show the median, interquartile range and observed range; points are individual months. The horizontal dashed line indicates the 60 mm monthly precipitation threshold used to classify a month as dry.

Monthly precipitation showed clear seasonality during the study period (June 2009–June 2010; Figure 1a; Table S1). Mean monthly rainfall was significantly lower during the dry season (9.63 ± 6.16 mm) than during the rainy season (146.59 ± 70.12 mm; Wilcoxon rank-sum test, W = 0, *p* = 0.0016). During the dry season, female proportions were similar between the canopy and understory, whereas during the rainy season the two strata diverged markedly, with females consistently more represented in the understory than in the canopy (Figure 1b).

**Figure 2:**
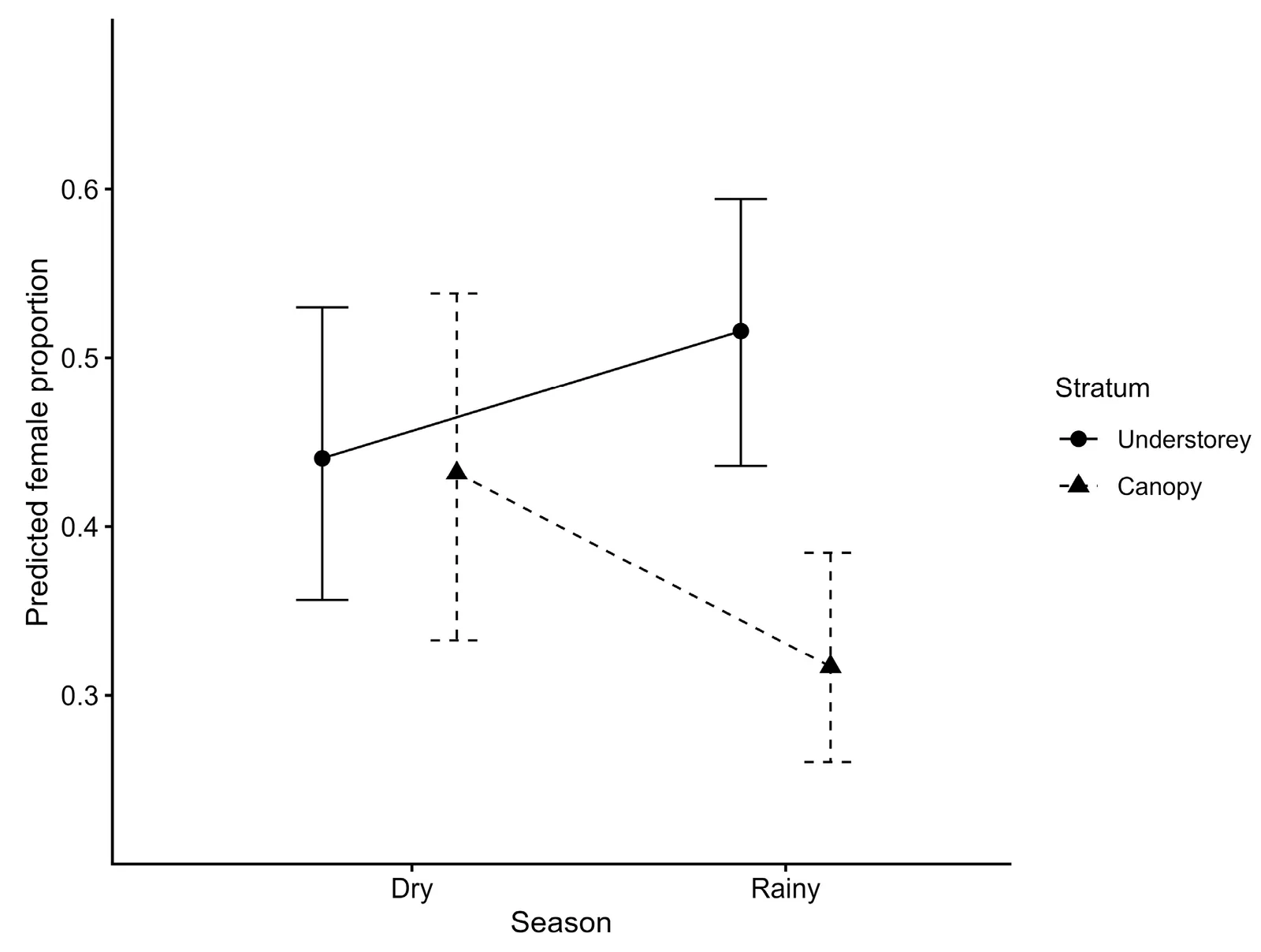
Predicted female proportion (±95% CI) of the ten most abundant fruit-feeding butterfly species from a binomial generalized linear mixed model including forest stratum (canopy vs. understorey) and season (dry vs. rainy) as fixed effects and species as a random effect. Solid lines and circles represent the understorey; dashed lines and triangles represent the canopy.

Overall abundance was higher in the canopy (64.7% of individuals among the ten focal species), but this pattern differed strikingly between seasons: in the dry season, most individuals were caught in the understory (66.2%), whereas during the rainy season most individuals were collected in the canopy (72.3%; χ^2^ = 220.79, df = 1, *p* < 0.001). Females were outnumbered by males in seven of the ten focal species, with female proportions ranging from 0.26 in *Fountainea ryphea* to 0.54 in *Colobura dirce* (Table 1). Sex-specific vertical stratification, based on total captures pooled across both seasons (Table 1), varied across species: three species—*Archaeoprepona demophon*, *Colobura dirce*, and *Fountainea ryphea*—had more females captured in the understory and more males captured in the canopy; three species—*Hamadryas amphichloe*, *Myscelia cyaniris*, and *Taygetis nympha*—had both sexes predominantly captured in the understory; and the remaining four species Smyrna blomfildia, Hamadryas amphinome, Historis odius, and *Historis acheronta* had both sexes predominantly captured in the canopy (Table 1). This classification is based on captures pooled across both seasons, whereas the seasonal interaction effect (Figure 2, Table 2) refers specifically to changes in the proportion of females within each stratum between the dry and rainy seasons. 

Our results revealed a seasonal pattern in the vertical sex distribution of butterflies. Analyses using both GLM and GLMM for the ten most abundant species showed that the main effect of forest stratum on female proportion was not significant when considered independently (GLM: *p* = 0.875; GLMM: *p* = 0.867). The main effect of season showed a marginal, borderline effect (GLM: *p* = 0.045; GLMM: *p* = 0.051). However, the interaction between stratum and season was significant (GLMM: OR = 0.44, 95% CI = 0.27–0.70, *p* < 0.001; GLM: OR = 0.43, 95% CI = 0.27–0.70, *p* < 0.001; Table 2), indicating that the relationship between female proportion and forest stratum varied seasonally. During the dry season, female proportions were similar in the canopy and understory (43% and 44%, respectively), whereas during the rainy season the difference between strata was more pronounced, with females proportionally more common in the understory than in the canopy (52% vs. 31%; Figure 2).

**Figure 3:**
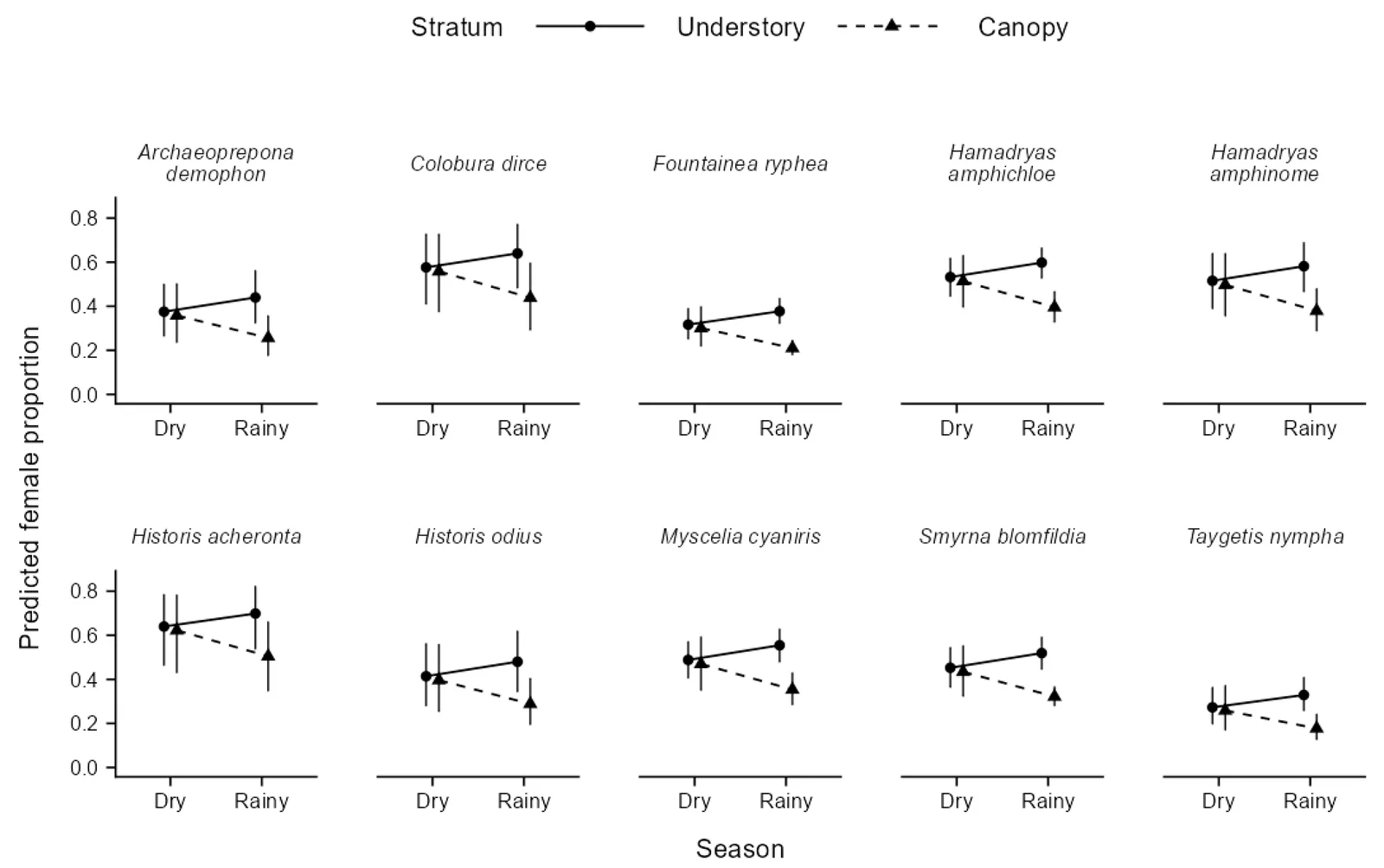
Predicted female proportion (±95% CI) of the ten most abundant fruit-feeding butterfly species across forest strata (canopy vs. understory) and seasons (dry vs. rainy). Solid lines and circles represent the understorey; dashed lines and triangles represent the canopy.

This general pattern was consistent across species-specific models, although significant effects were only detected for *C. dirce* (OR = 2.25, 95% CI = 1.04–4.88, *p* = 0.040), *H. acheronta* (OR = 2.92, 95% CI = 1.31–6.55, *p* = 0.009), and *H. amphichloe* (OR = 1.87, 95% CI = 1.11–3.19, *p* = 0.019) (Figure 3, Table 2).

## 4. Discussion

### 4.1. Seasonal Changes in Abundance and Vertical Stratification

We found a strong influence of seasonality on butterfly abundance and vertical stratification in the tropical dry forest of Bosque Seco Lalo Loor, with substantially higher abundance during the rainy season compared to the dry season. This pattern is consistent with previous studies on tropical insects, where precipitation-driven increases in resource availability are considered key drivers of population dynamics. This peak aligns with broader patterns observed in tropical dry forest insects, where the onset of rainfall reduces spatial variability in microclimate and stimulates the production of new foliage and fruits. These resources, which are severely limited during the prolonged dry season of deciduous forests, are critical for larval development and adult feeding, thereby driving rapid population growth during the rainy season [29]. This pronounced seasonal pattern in butterfly abundance has been documented across multiple years at this same site [39]. Similar patterns have been documented in Neotropical butterfly communities responding to environmental heterogeneity and seasonal changes in resource availability [14], although some studies report greater temporal stability in canopy assemblages [40].

In addition, we observed an overall seasonal shift in vertical distribution, with overall abundance higher in the understory during the dry season, and higher in the canopy during the rainy season. During the dry season, both males and females may avoid exposure to the increased temperature and reduced humidity of the canopy by occupying the understory, thereby reducing seasonal differences in sex-specific stratification [40]. Previous studies in Neotropical dry forests have shown that microclimatic variation strongly affects butterfly abundance, phenology, and community composition. During dry periods, butterflies frequently concentrate in cooler and more humid habitats such as riparian forests or dense understory vegetation [29]. Moreover, canopy environments may be several degrees warmer and substantially drier than the understory [14], generating physiologically stressful conditions that likely favor the use of understory refuges during the dry season. These initial results prompted us to examine the sex-specific dynamics of vertical distribution in greater detail.

### 4.2. Vertical Distribution of Males and Females and Its Relationship to Season

Our results reveal significant differences in the vertical distribution of male and female butterflies across seasons. As is typical in fruit-feeding assemblages, males generally outnumbered females [23], with females being less abundant in seven of the ten focal species (proportions ranging from 0.26 in *Fountainea ryphea* to 0.54 in *Colobura dirce*). Despite overall captures being concentrated in the canopy [6,41], three species (*Fountainea ryphea*, *Archaeoprepona demophon* and *Colobura dirce*) exhibited clear sex-specific stratification, with females predominating in the understory and males in the canopy. This aligns with studies demonstrating sex-specific vertical stratification in frugivorous butterflies [10], reflecting divergent reproductive strategies: males patrol the canopy to establish territories and intercept females [42], while females preferentially occur in the understory where suitable host plants grow [19].

Our results demonstrate significant differences in the vertical distribution of male and female Neotropical dry forest butterflies across seasons. While the sex ratio was similar in the understory and canopy in the dry season, likely due to environmental filtering forcing both sexes to seek the understory as a refuge [40], females were proportionally more abundant in the understory during the rainy season, suggesting a seasonal shift in female activity towards the understory and/or a shift in male activity towards the canopy. To the best of our knowledge, this is the first study documenting this specific interactive pattern in a tropical dry forest. Our analyses thus reveal that sex-specific responses contribute significantly to community-level patterns in vertical habitat use. To understand the drivers behind this seasonal divergence, we explored several behavioral and ecological mechanisms.

### 4.3. Behavioral and Ecological Mechanisms

One possible explanation for the observed seasonal shift in vertical sex distribution is related to oviposition behavior and host plant phenology. In many tropical forests, the rainy season is associated with peaks in vegetative growth, including the production of young leaves and new seedlings/saplings that are particularly suitable for herbivorous larvae. Because many butterfly species preferentially oviposit on young foliage, which is generally richer in nutrients and less tough than mature foliage [43], these seasonal leaf flushes may influence female oviposition activity [44]. Furthermore, suitable host plants are frequently concentrated in the understory and lower forest strata, where light conditions and plant composition favor the growth of shrubs, herbs, and saplings [45]. An increase in oviposition activity may therefore increase the concentration of females in the understory during the rainy season.

In contrast to the concentration in the understory of females that oviposit on plants near the ground, males of many species actively shift their activities toward the canopy during the rainy season. Indeed, our data indicate that the proportion of males in the canopy increases significantly during this period, suggesting that males are also actively moving into the upper stratum. In many butterfly species, mate-searching and territorial behaviors occur along flight corridors or sunlit patches within the canopy or midstory [10], and prominent landscape features often serve as critical rendezvous sites for encountering receptive females, a behavior widely documented as perching and hilltopping [20,42]. Studies on butterfly visual ecology suggest that males optimize female detection by positioning themselves above dark understory backgrounds while patrolling brighter canopy environments, a strategy that maximizes the visual contrast of flying females against the foliage below [46].

It is well established that flight morphology is associated with vertical stratification in tropical butterflies, with robust-bodied and fast-flying species more frequently associated with canopy habitats, whereas slower-flying species tend to occupy the understory [14,16]. Based on these ecomorphological traits, we would expect canopy-dwelling lineages, such as Charaxinae, to exhibit clear sex-specific vertical segregation. In alignment with this, our broad spatial data showed that large Charaxinae (*Archaeoprepona demophon* and *Fountainea ryphea)*, along with *Colobura dirce* (Nymphalinae), indeed exhibited the classic pattern of male prevalence in the canopy and female prevalence in the understory.

However, this ecomorphological pattern was not universal across all canopy-favoring species, nor was it static over time. For example, other canopy-dwelling species, such as *Historis acheronta* (Nymphalinae) and *Hamadryas amphichloe* (Biblidinae), did not show a strong baseline spatial segregation between sexes, but rather a significant seasonal shift in female proportions [6,47]. This suggests that vertical stratification is highly plastic and can be overridden by ecological constraints, such as the strict vertical distribution of specific larval host plants or localized resource availability [19,48]. Ultimately, while morphology sets the baseline for flight capabilities and broad spatial preferences, the differing reproductive priorities of male and female adult butterflies dictate their realized vertical and seasonal habitat use.

Diapause or diapause-like responses may reinforce these seasonal dynamics by reducing activity or delaying development during periods of unfavorable environmental conditions, particularly during the dry season [49,50]. In Neotropical butterflies, reproductive diapause during the dry season has been documented in several species, including *Catonephele acontius* [50]. Diapause presumably would not only affect female oviposition activities, but also male mate-locating activities, reducing both of these sex-specific activities during the dry season.

### 4.4. Species-Specific Responses and Conclusions

While our community-level analysis revealed a clear seasonal shift in vertical sex distribution, the strength of statistical support varied among species when tested individually. Although all ten focal species showed a qualitatively consistent pattern—an increase or stability in female proportion in the understory alongside a decrease in the canopy from the dry to the rainy season (Figure 3)—the stratum × season interaction was statistically significant in individual species-specific models only for *Colobura dirce*, *Historis acheronta*, and *Hamadryas amphichloe.* This variation underscores that vertical stratification is shaped not merely by broad environmental filtering, but also by intrinsic species traits [51], including ecomorphology, host plant ecology, and mating systems, which we explore below.

For example, species whose larval host plants are strictly distributed in the understory are expected to exhibit a stronger female bias in the lower stratum during the rainy season compared to species with generalist diets or canopy-distributed hosts [19]. However, even when host plants occur in both the understory and canopy, as with the *Cecropia* trees used by *Colobura dirce* and *Historis acheronta*, females may preferentially oviposit on understory seedlings and saplings, as reported for *C. dirce* [52]. Similarly, interspecific differences in male mate-locating strategies, whether patrolling canopy corridors or perching in understory sunflecks [42], will dictate male vertical distribution independently of broad seasonal trends. Based on our personal observation, *Historis acheronta* and *Colobura dirce*, at least, show a preference for perching in the midstorey and canopy.

These considerations suggest that host plant vertical distribution alone is unlikely to be the primary driver of seasonal shifts in sex ratio across vertical strata. Rather, a combination of mechanisms—including adult foraging ecology and fine-scale resource distribution—likely plays an important role. For instance, females might track seasonal shifts in fruit-fall at lower heights in the forest, or forage for specific reproductive resources. In a related fruit-feeding nymphalid [53], sustained female foraging activity was linked to the need for a continuous amino acid source for egg maturation, rather than to host plant location. Therefore, we interpret host plant specificity as a plausible, but not exclusive, factor driving species-specific vertical stratification.

In sum, we found that the rainy season in a tropical dry forest was associated with an increase in relative abundance of females in the understory and males in the canopy, suggesting seasonal shifts in vertical habitat use between sexes. Multiple mechanisms related to reproductive behavior, host plant phenology, microclimatic conditions, and sex-specific movement may contribute to these patterns. Future studies integrating experimental assessments of microclimate, host plant availability, and butterfly movement across forest strata would help clarify the processes underlying seasonal changes in vertical sex distribution. Together, our findings highlight the importance of considering sex-specific life histories, behavior and selective pressures in understanding spatial and temporal dynamics of butterfly communities in tropical dry forests.

## Figures and Tables

**Table 1:** Number of male and female individuals of the ten most abundant fruit feeding butterfly species captured in banana-bait traps across forest strata and seasons.

Species (Subfamily)	Stratum	Dry Females	Rainy Females	Dry Males	Rainy Males
*Fountainea ryphea* (Charaxinae)	Understory	33	75	38	53
*Fountainea ryphea* (Charaxinae)	Canopy	20	58	41	404
*Smyrna blomfildia* (Nymphalinae)	Understory	0	10	3	32
*Smyrna blomfildia* (Nymphalinae)	Canopy	9	138	15	253
*Hamadryas amphichloe* (Biblidinae)	Understory	22	53	30	43
*Hamadryas amphichloe* (Biblidinae)	Canopy	10	42	4	45
*Myscelia cyaniris* (Biblidinae)	Understory	31	40	47	40
*Myscelia cyaniris* (Biblidinae)	Canopy	3	33	5	25
*Taygetis nympha* (Satyrinae)	Understory	8	33	29	70
*Taygetis nympha* (Satyrinae)	Canopy	4	5	7	11
*Hamadryas amphinome* (Biblidinae)	Understory	1	3	1	4
*Hamadryas amphinome* (Biblidinae)	Canopy	3	32	2	51
*Archaeoprepona demophon* (Charaxinae)	Understory	12	4	11	7
*Archaeoprepona demophon* (Charaxinae)	Canopy	0	12	10	30
*Historis odius* (Nymphalinae)	Understory	3	0	0	0
*Historis odius* (Nymphalinae)	Canopy	3	15	3	46
*Colobura dirce* (Nymphalinae)	Understory	5	7	5	4
*Colobura dirce* (Nymphalinae)	Canopy	1	9	2	8
*Historis acheronta* (Nymphalinae)	Understory	1	1	0	0
*Historis acheronta* (Nymphalinae)	Canopy	1	16	0	18

**Table 2:** Effects of forest stratum, season, and their interaction on female proportion in fruit-feeding butterflies based on binomial GLM and GLMM analyses.

	GLM			GLMM		
Predictors	Odds Ratios	CI	*p*	Odds Ratios	CI	*p*
(Intercept)	0.60	0.36–0.99	0.044	0.78	0.55–1.13	0.189
Stratum	0.97	0.63–1.49	0.875	0.96	0.63–1.48	0.867
Season	1.37	1.01–1.88	0.045	1.36	1.00–1.85	0.051
*Colobura dirce*	2.25	1.04–4.88	0.040			
*Fountainea ryphea*	0.76	0.47–1.24	0.274			
*Hamadryas amphichloe*	1.87	1.11–3.19	0.019			
*Hamadryas amphinome*	1.76	0.95–3.30	0.074			
*Historis acheronta*	2.92	1.31–6.55	0.009			
*Historis odius*	1.16	0.57–2.31	0.680			
*Myscelia cyaniris*	1.57	0.93–2.70	0.098			
*Smyrna blomfildia*	1.34	0.81–2.24	0.260			
*Taygetis nympha*	0.61	0.34–1.09	0.094			
Stratum × Season	0.43	0.27–0.70	<0.001	0.44	0.27–0.7	<0.001
Random Effects						
σ2				0.06		
τ00				0.16		
ICC				0.72		
N				10		
Observations	39			39		
Marginal R2/Conditional R2	NA			0.300/0.806		

NA = not applicable (marginal and conditional R^2^ are calculated only for mixed models). Empty cells indicate that species were modeled as a random effect in the GLMM, so no species-level estimates are shown.

## Data Availability

Raw data is available upon request to the corresponding authors.

## Supplementary Materials

The following supporting information can be downloaded at https://www.mdpi.com/article/10.3390/insects17101053/s1. Table S1: Monthly precipitation recorded during the study period (June 2009–June 2010) based on CHIRPS data; Table S2: Annual precipitation in the study region from 2005 to 2015 based on CHIRPS data.

## Abbreviations

The following abbreviations are used in this manuscript:

BSLL	Bosque Seco Lalo Loor
CI	Confidence Interval
GLM	Generalized Linear Model
GLMM	Generalized Linear Mixed Model
OR	Odds Ratio
SD	Standard Deviation
