# Supplementary material for: Butterfly Female Proportion Increases in the Understorey During the Rainy Season in a Tropical Dry Forest
Source: Insects. 2026 Oct 13;17(10):1053. doi: 10.3390/insects17101053 (PMC13705022; doi:10.3390/insects17101053)
Supplement: Supplementary file 1 [file insects-17-01053-s001.zip › insects-4527966-supplementary.pdf]

Supplementary Materials

Table S1. Monthly precipitation recorded during the study period (June 2009–June 2010) based on CHIRPS data.

Month	Total precipitation (mm)	Mean daily precipitation (mm)	Days with data
2009-06	11.875	0.396	30
2009-07	12.218	0.394	31
2009-08	6.135	0.198	31
2009-09	6.528	0.218	30
2009-10	6.081	0.196	31
2009-11	0.000	0.000	30
2009-12	14.357	0.463	31
2010-01	107.386	3.464	31
2010-02	218.250	7.795	28
2010-03	226.307	7.300	31
2010-04	103.71	3.46	30
2010-05	77.307	2.494	31
2010-06	19.835	0.661	30

Table S2. Annual precipitation in the study region from 2005 to 2015 based on CHIRPS data.

Year	Annual precipitation (mm)
2005	721.755
2006	765.660
2007	850.779
2008	1057.507
2009	553.128
2010	869.834
2011	678.824
2012	1167.007
2013	1124.261
2014	857.775
2015	840.727
